# Effectiveness of Organized Strategies to Enhance Access to Preventive Oral Cancer Screening Among High‐Risk Individuals in Brazil: A Randomized Clinical Trial in Primary Care

**DOI:** 10.1002/ijc.70511

**Published:** 2026-04-21

**Authors:** Marcia Frias Pinto Marinho, Keith Bullia da Fonseca Simas, Patrícia Vinas Heras, Claudia Aparecida Serra da Cruz, Amanda Martins dos Santos Bessa, Mário José Romãnach, Aline Corrêa Abrahão, Maria Augusta Visconti, Michelle Agostini

**Affiliations:** ^1^ School of Dentistry Federal University of Rio de Janeiro Rio de Janeiro Brazil; ^2^ Municipal Health Department of Rio de Janeiro Rio de Janeiro Brazil

**Keywords:** early diagnosis, oral cancer, public health, screening

## Abstract

This randomized clinical trial assessed the performance of organized, risk‐based oral cancer (OC) screening strategies compared with the conventional opportunistic approach. Conducted from December 2023 to December 2024 across 35 primary health care (PHC) units in Rio de Janeiro, Brazil, the study included adults aged ≥ 35 years identified as tobacco users in the public electronic medical record system and considered high‐risk for preventive oral examination (POE). PHC units were allocated to three groups: control (opportunistic screening without active invitation; 12 units), experimental I (organized invitation for POE plus home‐visit support; 11 units), and experimental II (organized invitation for POE plus an awareness campaign; 12 units). The 23 PHC units implementing organized screening included 2735 registered tobacco users, whereas the 12 units maintaining opportunistic screening included 1048. Consequently, POE coverage reached 77.2% in the organized screening arms versus 3.6% under opportunistic screening (*p* < 0.05). POE adherence was higher with organized invitation plus community awareness (77.2%) than with home‐visit support (58.1%). A total of 15 biopsies were performed within the organized screening groups, identifying 6 cases of oral potentially malignant disorders and 5 cases of OC. In contrast, biopsies conducted outside the screening program demonstrated significantly lower detection rates (*p* < 0.0001). These findings demonstrate that organized, invitation‐based screening integrated into PHC can substantially expand access to early diagnosis among high‐risk and socially vulnerable individuals who would otherwise remain unscreened, reinforcing PHC as a strategic and equitable setting for sustainable early OC detection.

## Abstract (highlights)

What's new?

This randomized clinical trial evaluated the performance of organized risk‐based strategies to expand access to cancer preventive oral examination among vulnerable populations within the public health system of Rio de Janeiro, Brazil. Compared with an opportunistic approach, the organized invitation‐based oral cancer screening approaches systematically targeting high‐risk individuals within primary healthcare centers expanded screening coverage and increased the detection of malignant and potentially malignant oral lesions. With no additional workforce required and minimal disruption to existing workflows, the model proved sustainable within routine primary healthcare services and may represent a scalable strategy to improve access to early oral cancer diagnosis.

## Graphical abstract

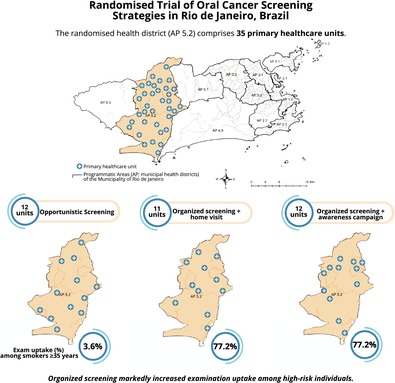

**Abbreviations**
CG: control group
EG: experimental group
FO‐UFRJ: School of Dentistry of the Federal University of Rio de Janeiro
OC: oral cancer
PHC: primary health care
PMOD: potentially malignant oral disorder
POE: preventive oral examination
SMS‐RJ: Municipal Health Department of Rio de Janeiro

## 1 Introduction

Oral cancer (OC) remains a major public health challenge, characterized by high morbidity and mortality rates and a predominance of late‐stage diagnoses, particularly in low‐ and middle‐income countries such as Brazil [1, 2]. In Brazil, the current strategy for early detection of OC is primarily based on opportunistic screening; however, despite decades of implementation, most cases continue to be diagnosed at advanced stages [3, 4]. From a sociodemographic perspective, alcohol and tobacco consumption are most prevalent among socially and economically vulnerable groups, who also face substantial barriers to accessing health services, ultimately contributing to delayed diagnosis [5, 6].

In this context, implementing effective early detection strategies becomes even more urgent. Evidence from the landmark Kerala trial in India, one of the largest population‐based studies on OC screening, demonstrated that visual examination performed by trained health workers significantly reduces OC mortality among individuals with high‐risk behaviors, such as tobacco and alcohol use [7, 8]. Consistent with these findings, the IARC Handbooks recognize visual screening targeted at high‐risk groups as an evidence‐based and cost‐effective intervention for reducing OC mortality [9, 10, 11]. These converging lines of evidence support organized, risk‐based screening as a strategy that favors the early detection of OC and potentially malignant oral disorders (PMOD), particularly when integrated with timely diagnosis and treatment within primary health care (PHC) networks [12, 13, 14].

This study evaluated the performance of different strategies to expand access to preventive oral examination (POE) among vulnerable populations within the public health system of Rio de Janeiro, Brazil.

## 2 Methodology

**FIGURE 1:**
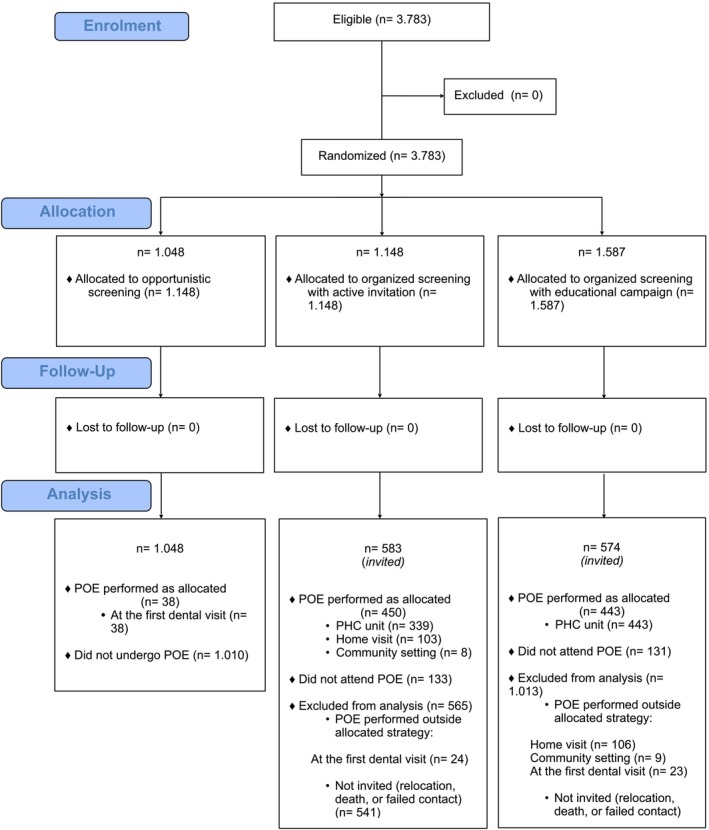
CONSORT flow diagram of the study.

This randomized clinical trial was conducted by the School of Dentistry of the Federal University of Rio de Janeiro (FO‐UFRJ), in partnership with the Municipal Health Department of Rio de Janeiro (SMS‐RJ). The trial was conducted between January and December 2024, in accordance with the CONSORT guidelines (Figure 1), in one randomly selected regional health district of Rio de Janeiro, comprising 35 PHC units.

The at‐risk population was defined as individuals aged 35 years or older with an active smoking history documented in PHC electronic health records. The sample was selected by convenience, based on all eligible individuals registered as of November 2023. The 35 PHC units were randomly allocated into three groups: a control group (CG), an experimental group I (EGI), and an experimental group II (EGII). Each implemented a different strategy for organizing the workflow for early detection of OC within PHC, intending to compare their effectiveness in expanding access to the POE among the target population. Randomization at the PHC unit level was adopted to minimize contamination between participants within the same service environment, as the intervention involved workflow reorganization and team‐level engagement. However, the primary outcomes were defined and measured at the individual level, and analyses were conducted accordingly, consistent with the predefined analytical plan. The trial was therefore designed as a randomized allocation of PHC units with individual‐level outcome assessment and was not primarily powered or structured as a classical cluster‐randomized trial with hierarchical modeling.

The analyzed sample ensured a statistical power of 99% (*β* = 0.01) to detect medium effect sizes (*w* = 0.30) in analyses of adherence and POE uptake, with a significance level of 5% (*α* = 0.05). Sample size calculations were performed using G*Power software, based on the parameters proposed by Cohen [15]. The implementation of the experimental group's strategies, which involved organized screening through the active recruitment of smokers and systematic provision of POE, was supported by 496 PHC professionals for the recruitment stage, including community health workers and Oral Health Assistants. A total of 169 professionals, including dentists, physicians, and nurses, participated in the performance of POE. Recruitment activities and POE procedures were recorded using a structured electronic form (Google Forms), which the professionals themselves completed. Data were consolidated in real‐time through dynamic dashboards developed on the Microsoft Power BI platform (Microsoft Corporation) and cross‐checked against the user database extracted from the electronic health record system.

All participating professionals underwent pre‐intervention operational training, with technical support provided to ensure standardized implementation of the intervention. The strategies between the experimental groups began to diverge in November 2024: EGI implemented active case finding in the community, including home visits to users who did not respond to the invitation for POE, whereas EGII conducted a prevention campaign throughout the same month, aimed at raising public awareness about OC and increasing the availability of POE. The EGII campaign, entitled “Open Your Mouth,” was implemented following a structured alignment videoconference conducted in real time and simultaneously with all PHC teams in the group. The session was scheduled during routine team meeting hours to avoid disruptions to service logistics and to ensure the participation of all professionals. The campaign included: (1) dissemination of standardized educational materials (posters displayed in PHC units and campaign‐branded T‐shirts worn by staff); (2) intensified outreach activities within PHC units and surrounding community spaces; (3) integration with existing local programs, such as tobacco control and physical activity initiatives; (4) engagement of adolescents as peer educators in awareness activities; and (5) visible institutional endorsement by local health authorities. These components were implemented concurrently during the intervention period to promote POE attendance.

In the CG, no additional intervention or standardization was introduced, and the existing opportunistic screening strategy was maintained. Given the nature of the intervention, which involved reorganization of service practices, blinding of participants was not feasible.

Descriptive and comparative statistical analyses were conducted to evaluate differences among groups. The primary outcome was the number of POE performed in registered smokers per group. Secondary outcomes included the number of biopsies performed and the diagnoses of PMOD or OC. Categorical variables included sex, age group, professional performing the POE, setting of the examination (within PHC units or through home and community outreach), month of examination, referral and attendance at specialist care, and biopsy outcomes. These variables were summarized as absolute and relative frequencies. Numerical variables, such as the number of invitations per participant and the interval between referral and specialist care, were described using measures of central tendency and dispersion and compared across groups with the nonparametric Mann–Whitney test. Categorical variables were analyzed using chi‐squared or Fisher's exact tests, as appropriate. All analyses were conducted in R (R Core Team, 2025), with a two‐sided significance level set at 5% (*p* < 0.05).

All eligible participants remained under systematic monitoring throughout the intervention period (January to December 2024), allowing complete ascertainment of study outcomes. Participants who underwent POE outside their allocated strategy, relocated outside the catchment area, or died were excluded from the analytical sample to preserve group allocation consistency (Figure 1) and were treated as analytical exclusions rather than losses to follow‐up.

## 3 Results

A total of 3783 smokers registered across the 35 participating PHC units were identified and included according to the allocation of their respective units. There were no statistically significant differences between groups in sex or age distribution (*p* > 0.05). Overall, 58.7% of participants were women and 41.3% were men (Table 1).

**TABLE 1:** Distribution of at‐risk participants across 35 PHC units, according to study group, sex, and age group.

Variable	Category	Total	Group			*p*
			CG	EGI	EGII	
			Frequency (%)			
High‐risk individuals	—	3.783 (100.0%)	1.048 (27.7%)	1.148 (30.3%)	1.587 (42.0%)	—
PHC units	—	35 (100.0%)	12 (34.3%)	11 (31.4%)	12 (34.3%)	—
Sex	Female	2220 (58.7%)	629 (60.0%)	678 (59.1%)	913 (57.5%)	0.4255
	Male	1563 (41.3%)	419 (40.0%)	470 (40.9%)	674 (42.5%)	
Age group (years)	35–44	582 (15.4%)	148 (14.1%)	172 (15.0%)	262 (16.5%)	0.4782
	45–54	796 (21.0%)	228 (21.8%)	251 (21.9%)	317 (20.0%)	
	55–64	1171 (31.0%)	345 (32.9%)	351 (30.6%)	475 (29.9%)	
	65–74	1000 (26.4%)	263 (25.1%)	300 (26.1%)	437 (27.5%)	
	≥ 75	234 (6.2%)	64 (6.1%)	74 (6.4%)	96 (6.0%)	

Abbreviation: PHC, primary health care.

A statistically significant association was found between group allocation and adherence to invitation, with higher attendance in EGII compared to EGI. Additionally, there was a significant association between the allocation group and the number of POE performed, which was significantly greater in both experimental groups than in the CG (*p* < 0.05) (Table 2). Each participant received between one and seven invitation attempts to attend the PHC unit for POE. In EGI, most participants responded to the first call (64.6%), while 33.0% required two attempts and 2.4% required three. In EGII, 51.0% adhered after the first invitation, 37.7% after the second, and 8.6% after the third, with a small proportion requiring up to seven invitation attempts. The mean number of invitation attempts was 1.4 in EGI and 1.7 in EGII (*p* > 0.05).

**TABLE 2:** Distribution of participants with an active ICD code for tobacco use (smokers), invited for screening and examined through preventive oral examination (POE), by study group.

Group	Individuals with an active ICD code	Individuals invited for POE	Individuals attended POE (at PHC unit)	POE performed as allocated
			Frequency (%)	Frequency (%)
CG	1048	—	—	38 (3.6%^b^)
EGI	1148	583	339 (58.1%^a^)	450 (77.2%^a^)
EGII	1587	574	443 (77.2%^a^)	443 (77.2%^a^)
*p* value	—	—	< 0.0001	< 0.0001

Abbreviations: ICD, international classification of diseases; PHC, primary health care; POE, preventive oral examination.

^a^ Percentage relative to the total number of individuals invited through the screening program.

^b^ Percentage relative to the total sample.

In EGI, 58.1% of invited participants attended the PHC for POE, whereas in EGII this proportion was higher, reaching 77.2%. Considering only participants who underwent POE according to their assigned strategy, 3.6% in the CG (opportunistic screening), 58.1% in EGI (screening within PHC units and through home visits), and 77.2% in EGII (screening within PHC units supported by a community campaign in November) completed the examination. EGII achieved higher adherence without relying on home visits, which were necessary in EGI to reach comparable coverage (Table 2).

**FIGURE 2:**
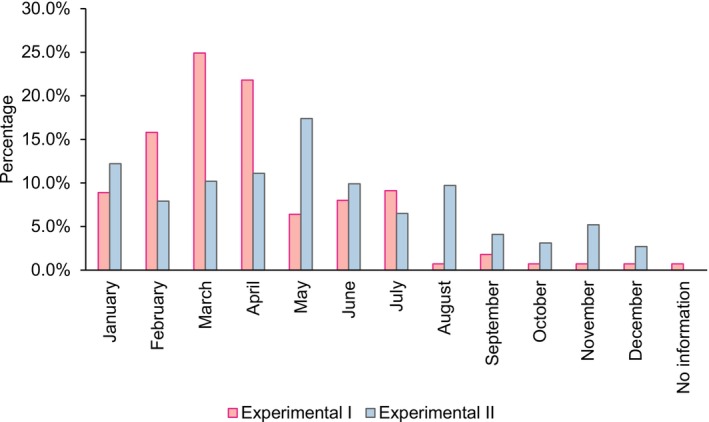
Monthly distribution of preventive oral cancer screening performed in experimental groups I and II during the screening period.

POE was performed primarily by dentists (EGI: 96.2%; EGII: 96.8%) with occasional participation of physicians and nurses, who supported the interventions but demonstrated limited adherence to oral screening. In CG, 38 POE were performed under the routine opportunistic care model, with no subsequent referrals to stomatology or biopsies recorded. In EGI, which combined organized screening with active outreach, 75.3% of POE were conducted in PHC units, 22.9% during home visits, and 1.8% in community settings. A statistically significant difference was observed in the monthly distribution of POE between the experimental groups (*p* < 0.05) (Figure 2). In EGI, the highest concentration occurred in March (24.9%) and April (21.8%), whereas in EGII, the peak was in May (17.4%). In both experimental groups, a downward trend was observed over time, except for a renewed increase in EGII during the prevention campaign in November, when 5.2% of POE were performed. In contrast, only three POE (0.7%) were conducted in EGI in the same period.

Among participants examined during POE, 2.7% in EGI (12/450) and 8.6% in EGII (38/443) were referred for specialist evaluation (*p* < 0.05). Of those referred, 50.0% (6/12) in EGI and 39.5% (15/38) in EGII attended their specialist appointment (*p* > 0.05). Biopsies were performed in 66.7% (4/6) of EGI and 73.3% (11/15) of EGII participants who attended the specialist appointment (*p* > 0.05). Among biopsied cases, PMOD were identified in 0.0% (0/4) of EGI and 54.5% (6/11) of EGII, while OC was diagnosed in 75.0% (3/4) and 18.2% (2/11), respectively (*p* > 0.05). Benign lesions accounted for 25.0% (1/4) of biopsies in EGI and 27.3% (3/11) in EGII (Table 3). Among the six PMOD cases, two occurred in women and four in men, all aged > 60 years. All OC cases were histologically confirmed. Four were squamous cell carcinomas diagnosed in men over 50 years of age: two in the floor of the mouth (3 and 4 cm in size), one in the lateral border of the oral tongue (5 cm), and one at the base of the tongue (oropharynx, 3 cm). The fifth case corresponded to a secretory carcinoma, a rare salivary gland malignancy and not typically associated with tobacco exposure, diagnosed in a 35‐year‐old man and located in the buccal mucosa. No OC cases were identified in the CG. Tumor staging information was not available within the scope of this study, as patients were referred to specialized oncology hospitals for staging and treatment following histopathological diagnosis, and access to hospital medical records was not obtained.

**TABLE 3:** Distribution of referrals and biopsies following POE among tobacco users in PHC units, by study group.

Group	POE	Referred to the stomatologist	Attended the stomatology appointment	Number of biopsies performed	PMOD cases	OC cases	Benign lesions
		*n* (%)^a^	*n* (%)^b^	*n* (%)^c^	*n* (%)^d^		
CG	38	0 (0.0%)	0 (‐)	0 (‐)	0 (‐)	0 (‐)	0 (‐)
EGI	450	12 (2.7%)	6 (50.0%)	4 (66.7%)	0 (0.0%)	3 (75.0%)	1 (25.0%)
EGII	443	38 (8.6%)	15 (39.5%)	11 (73.3%)	6 (54.5%)	2 (18.2%)	3 (27.3%)
*p* value^e^		0.0001	0.5195	1.0000	0.0769		

Abbreviations: PHC, primary health care; PMOD, potentially malignant oral disorders; POE, preventive oral examination.

^a^ Percentage calculated using the total number of POE performed in each study group as the denominator.

^b^ Percentage calculated using the total number of referrals to the stomatologist in each study group as the denominator.

^c^ Percentage calculated using the total number of participants who attended the stomatology appointment in each study group as the denominator.

^d^ Percentage calculated using the total number of biopsies performed in each study group as the denominator.

^e^ *p* values were calculated for comparisons between experimental groups (EGI vs. EGII).

Of the 140 biopsies performed in the selected health district in 2024, 15 were conducted in participants from the organized screening groups. Among these, 6 cases of PMOD (40.0%) and 5 cases of OC (33.3%) were identified. In contrast, among the 125 biopsies performed in routine or emergency care, 17 (13.6%) resulted in a diagnosis of PMOD and 6 (4.8%) in OC. The difference in diagnostic yield between screening and non‐screening biopsies was statistically significant (*p* < 0.0001). Benign lesions accounted for 102 of 125 (81.6%) biopsies performed in routine or emergency care, compared with 4 of 15 (26.7%) in the screening groups, highlighting a greater proportion of potentially malignant or malignant findings in the high‐risk population.

## 4 Discussion

Consistent with evidence showing that targeted screening of high‐risk individuals is an effective strategy for reducing cancer mortality [7, 8, 9, 10, 11, 12, 13, 14], this study implemented an organized screening program for the experimental groups, fully integrated into the logic of PHC. The strategy proved effective in significantly expanding access to POE among vulnerable populations typically underserved in conventional models of early cancer detection [16]. With no statistically significant differences in POE coverage between the experimental groups, both the active community outreach model (EGI) and the prevention campaign model (EGII) demonstrated similar potential to reach high‐risk individuals.

Based on risk profiles available in PHC information systems, individuals with an active history of tobacco use, the leading risk factor for developing OC [17, 18], were defined as the priority population for screening. The ability to identify this group within PHC enabled systematic monitoring of higher‐risk individuals using routinely collected data. In the studied territory, approximately 75% of the population is registered in the PHC system, thereby strengthening the representativeness of this strategy in the local context. Nevertheless, reliance on electronic health record documentation has inherent limitations, as not all smokers are registered in public information systems, and underreporting remains common. Some individuals may be covered exclusively by private health insurance, while others live in conditions of extreme social vulnerability without consistent access to public health services [19, 20]. Therefore, the findings primarily reflect the effectiveness of organized screening among registered high‐risk individuals rather than the entire at‐risk population in the community.

Although global smoking prevalence declined between 2000 and 2020, Brazil recorded its first increase in adult smoker prevalence in 2024, with higher rates among men [21]. In this study, most screened individuals were women, possibly reflecting differences in health‐seeking behavior and access to services [22]. These findings highlight the need for targeted and equity‐oriented strategies to identify and engage high‐risk groups.

A key limitation was the inability to systematically identify alcohol users in PHC information systems, despite alcohol consumption being a well‐established risk factor for OC and other noncommunicable diseases. The inability to incorporate alcohol consumption as an active screening criterion is particularly concerning, given the synergistic effect of alcohol and tobacco on the risk of developing OC [14].

PHC professionals are uniquely positioned to identify social determinants and risk factors, foster engagement in health initiatives, and help overcome barriers related to misinformation, stigma, and communication challenges around cancer. Within this framework, community health workers proved essential for recruiting high‐risk individuals who would likely remain unreached through routine dental care [23, 24]. In this study, the EGII model, combining organized screening with a community prevention campaign, achieved higher appointment adherence, reflecting the combined effect of structured invitation and additional community engagement strategies, which may enhance feasibility and scalability in settings with limited human resources.

The adoption of scheduled invitation systems, supported by digital technologies and combined with targeted outreach to high‐risk populations, can expand the coverage of organized screening and contribute to reducing inequities in access to POE. Equally important is the greater involvement of physicians and nurses, given that high‐risk individuals often do not directly seek dental services and therefore are not examined in dental clinics. Despite their inclusion in the study, physicians and nurses did not actively participate in screening. These results highlight the need to develop targeted engagement strategies for these professionals, including the implementation of clear and operational clinical guidelines that systematically incorporate POE for high‐risk groups. Strengthening shared clinical responsibility across professional categories may be critical to achieving sustained screening coverage within routine practice, and, within this framework, the structured identification of high‐risk smokers in PHC may also facilitate integrated risk‐based prevention strategies, including systematic assessment for symptoms suggestive of laryngeal cancer and, where available and feasible, alignment with established lung cancer screening protocols.

The results also revealed marked seasonality in the implementation of scheduled screening activities, with higher concentrations observed during the months when research supporters visited PHC units to provide guidance on work processes and encourage local initiatives. This pattern indicates that implementation was strongly influenced by prior team mobilization and structured invitation processes, particularly the active engagement of community health workers. While the community campaign in EGII coincided with a modest increase in preventive examinations, its effect appears to have been complementary and time‐limited rather than the primary driver of participation, especially when compared with structured invitation and active outreach mechanisms. To sustain performance under routine conditions, ongoing institutional reinforcement may be necessary, including the incorporation of POE indicators into official monitoring systems.

Incorporating OC screening into the routine activities of PHC units for at‐risk individuals remains a challenge, as demonstrated by the progressive decline in screening numbers over time, even among groups undergoing organized screening. This finding highlights the need for well‐defined institutional protocols, supported by continuous education strategies, as practical and sustainable measures to promote early detection and reduce inequities in access to diagnosis.

OC screening followed by biopsy in suspected cases remains the gold standard for identifying PMOD and early‐stage malignant lesions, focused on early detection [25, 26, 27, 28]. In 2024, a total of 140 biopsies were performed in the health district where the study was implemented, of which 15 were conducted among screening participants and 125 among individuals not included in the organized screening. Although the number of biopsies and cases detected during screening was limited, statistically significant differences in diagnostic yield were observed between strategies. Among the 15 biopsies performed following organized screening, 6 cases of PMOD and 5 cases of OC were identified, all squamous cell carcinomas in men over 50 years of age, except for one case of secretory carcinoma in a 35‐year‐old man. These findings should be interpreted considering the study's primary objective. Rather than conducting a formal cost‐effectiveness analysis, this trial aimed to assess whether organized, invitation‐based screening could expand access to POE among high‐risk individuals who would otherwise remain unscreened. Indeed, the individuals diagnosed with squamous cell carcinoma already had lesions 3 cm or larger and had not sought medical attention before being invited for the examination. Screening coverage increased substantially from 3.6% in the CG to 77.2% in the organized arms, representing a meaningful gain in equitable access. The strategy was implemented within the existing primary care infrastructure, without additional hiring or parallel services, relying instead on workflow reorganization, targeted invitation through electronic medical records, and structured attendance monitoring, suggesting limited incremental costs.

By contrast, among biopsies performed in routine or emergency care among individuals outside the screening eligibility group (including non‐smokers and those younger than 35 years), diagnostic rates were considerably lower (13.6% for PMOD and 4.8% for OC), with benign lesions predominating (81.6%). These findings demonstrate that active screening in high‐risk groups enhances diagnostic accuracy for suspicious lesions and facilitates more targeted referrals, and over time may contribute to earlier detection and improved clinical outcomes.

However, the study also revealed a critical barrier in the care pathway: only 50.0% (6/12) in EGI and 39.5% (15/38) in EGII attended specialist evaluation in oral medicine after referral. Although appointments were guaranteed, low adherence likely reflects geographic and socioeconomic barriers, including transportation costs, competing daily priorities, and difficulties reaching referral centers located outside patients' immediate catchment areas. Fear of diagnosis may also have contributed [5]. These findings indicate that expanding screening alone is insufficient if continuity of care remains constrained. Effective integration of referral pathways and timely diagnostic resolution represents a critical determinant of the overall impact of organized screening programs. Strengthening primary care capacity through training general dentists to perform simple biopsies and incorporating teleconsultation support may help reduce geographic barriers and improve timely diagnostic resolution among vulnerable populations.

Similar findings have been reported in other organized OC screening initiatives. Community‐based screening programs in Thailand and São Paulo, Brazil, have shown that targeted screening of high‐risk populations increases the detection of PMOD and OC compared with routine care [29, 30, 31]. The Barretos program, likewise, conducted in Brazil, reported overall detection rates of approximately 1.07 OC and 1.17 PMOD per 1000 examinations over 7 years, with variability across implementation phases [13], with PMOD rates of approximately 0.6 in 2017 and 2020, and OC rates around 0.7 in 2014 and 2017. In the present study, conducted during the first year of organized screening within PHC, detection rates were 0.56 OC and 0.67 PMOD per 1000 examinations, values comparable to those reported in specific years of the Barretos program. As described by Vazquez et al., organized screening initiatives often exhibit a learning curve, characterized by progressive refinement of referral criteria, reduction of unnecessary biopsies, and increasing diagnostic precision over time.

In line with international recommendations highlighting the heterogeneity of early OC detection strategies and the need for sustainable, systematic, and equity‐oriented actions targeting vulnerable populations [32], the present findings reinforce the strategic importance of embedding organized screening within PHC to expand access to POE and enable timely diagnosis of suspicious lesions. Mobilizing public health managers to incorporate structured, equity‐oriented screening strategies into national and local cancer control plans is essential to reduce advanced‐stage diagnoses, strengthen continuity of care, and ultimately improve morbidity and mortality outcomes associated with OC [33, 34, 35].

## 5 Conclusion

The implementation of organized screening models targeting high‐risk populations confirmed the hypothesis that these strategies are more effective than the opportunistic approach in expanding access to POE. Interventions involving home visits and community campaigns successfully reached individuals who would otherwise be unlikely to undergo screening in conventional routine care, thereby reducing access barriers. Moreover, referrals generated through organized screening demonstrated greater diagnostic effectiveness, with higher positivity rates for PMOD and OC compared to the opportunistic model.

These findings reinforce PHC as a strategic setting for sustainable early detection initiatives and highlight the potential of organized screening to reduce inequities in access to cancer diagnosis. The evidence generated can inform replicable public policies in similar contexts, particularly when supported by standardized protocols and data‐driven tools that enhance the scalability, monitoring, and sustainability of organized screening, contributing to earlier detection and improved cancer outcomes.

## Author Contributions

**Marcia Frias Pinto Marinho:** conceptualization, methodology, investigation, data curation, formal analysis, project administration, supervision, writing – original draft, writing – review and editing, visualization. **Keith Bullia da Fonseca Simas:** investigation, data curation, writing – review and editing. **Patrícia Vinas Heras:** investigation, writing – review and editing, data curation. **Claudia Aparecida Serra da Cruz:** investigation, writing – review and editing, data curation. **Amanda Martins dos Santos Bessa:** investigation, writing – review and editing, data curation. **Mário José Romãnach:** investigation, writing – review and editing. **Aline Corrêa Abrahão:** investigation, writing – review and editing. **Maria Augusta Visconti:** conceptualization, methodology, writing – review and editing, formal analysis, supervision. **Michelle Agostini:** conceptualization, methodology, formal analysis, funding acquisition, writing – review and editing, supervision.

## Funding

This study was financially supported by public funding agencies, including the Conselho Nacional de Desenvolvimento Científico e Tecnológico/National Council for Scientific and Technological Development (CNPq, grant No. 420633/2023‐5) and the Fundação Carlos Chagas Filho de Amparo à Pesquisa do Estado do Rio de Janeiro/Carlos Chagas Filho Foundation for Research Support of the State of Rio de Janeiro (FAPERJ, grant No. E‐26/210.635/2024). The Article Processing Charge (APC) for the publication of this research was funded by the Coordenação de Aperfeiçoamento de Pessoal de Nível Superior/Coordination for the Improvement of Higher Education Personnel (CAPES) (ROR identifier: 00x0ma614). For open access, the authors have applied a Creative Commons Attribution (CC BY) license to any accepted version of the manuscript.

## Disclosure

This manuscript is part of the first author's doctoral dissertation. The funding agencies had no role in the study design; data collection, analysis, or interpretation; writing of the manuscript; or the decision to submit the article for publication.

## Ethics Statement

The study was conducted in accordance with the Declaration of Helsinki and was approved by the Research Ethics Committees of the School of Dentistry of the Federal University of Rio de Janeiro (approval No. 6.030.135) and the Municipal Health Department of Rio de Janeiro (approval No. 6.390.106). The trial was prospectively registered on ClinicalTrials.gov (NCT06231537). All participants provided informed consent prior to participation.

## Conflicts of Interest

The authors declare no conflicts of interest.

## Data Availability

The data that support the findings of this study are available from the corresponding author upon reasonable request.
